# Supplementary material for: Geographical Disparities in Esophageal Cancer Incidence and Mortality in the United States
Source: Healthcare (Basel). 2023 Feb 25;11(5):685. doi: 10.3390/healthcare11050685 (PMC10001323; doi:10.3390/healthcare11050685)
Supplement: Supplementary file 1 [file healthcare-11-00685-s001.zip › healthcare-2182534-supplementary.pdf]

Supplemental Material

Vedire Y., Groman A., Siromoni B., Yendamuri S., Mukherjee S, Geographical Disparities in Trends of Esophageal Cancer Incidence and Mortality between 1975 and 2016

Content

1. Table S1. STROBE Statement—Checklist of items that should be included in reports of cohort studies
2. Table S2. Age adjusted incidence and mortality rates of esophageal cancer patients between 1975 and 2016
3. Table S3. Attributable risk percentage and population attributable risk percent between 1975 and 2016 for esophageal cancer incidence.
4. Figure S1. Kaplan Meier Survival Plot for Overall Survival
5. Figure S2. Kaplan Meier Survival Plot for Disease Specific Survival

Table S1. STROBE Statement—Checklist of items that should be included in reports of cohort studies

	Item No	Recommendation	Page No[a]
**Title and abstract**	1	(*a*) Indicate the study's design with a commonly used term in the title or the abstract	2
		(*b*) Provide in the abstract an informative and balanced summary of what was done and what was found	2
**Introduction**			
Background/rationale	2	Explain the scientific background and rationale for the investigation being reported	3
Objectives	3	State specific objectives, including any prespecified hypotheses	3
**Methods**			
Study design	4	Present key elements of study design early in the paper	4
Setting	5	Describe the setting, locations, and relevant dates, including periods of recruitment, exposure, follow-up, and data collection	4
Participants	6	(*a*) Give the eligibility criteria, and the sources and methods of selection of participants. Describe methods of follow-up	4, 5
		(*b*) For matched studies, give matching criteria and number of exposed and unexposed	N/A
Variables	7	Clearly define all outcomes, exposures, predictors, potential confounders, and effect modifiers. Give diagnostic criteria, if applicable	5
Data sources/ measurement	8*	For each variable of interest, give sources of data and details of methods of assessment (measurement). Describe comparability of assessment methods if there is more than one group	4, 5
Bias	9	Describe any efforts to address potential sources of bias	5
Study size	10	Explain how the study size was arrived at	N/A
Quantitative variables	11	Explain how quantitative variables were handled in the analyses. If applicable, describe which groupings were chosen and why	5
Statistical methods	12	(*a*) Describe all statistical methods, including those used to control for confounding	5
		(*b*) Describe any methods used to examine subgroups and interactions	N/A
		(*c*) Explain how missing data were addressed	5
		(*d*) If applicable, explain how loss to follow-up was addressed	N/A
		(*e*) Describe any sensitivity analyses	N/A
**Results**			
Participants	13*	(a) Report numbers of individuals at each stage of study—eg numbers potentially eligible, examined for eligibility, confirmed eligible, included in the study, completing follow-up, and analysed	6, 7
		(b) Give reasons for non-participation at each stage	N/A

		(c) Consider use of a flow diagram	N/A
Descriptive data	14*	(a) Give characteristics of study participants (eg demographic, clinical, social) and information on exposures and potential confounders	6, 7
		(b) Indicate number of participants with missing data for each variable of interest	6, 7
		(c) Summarise follow-up time (eg, average and total amount)	N/A
Outcome data	15*	Report numbers of outcome events or summary measures over time	6, 8
Main results	16	(*a*) Give unadjusted estimates and, if applicable, confounder-adjusted estimates and their precision (eg, 95% confidence interval). Make clear which confounders were adjusted for and why they were included	7, 8
		(*b*) Report category boundaries when continuous variables were categorized	N/A
		(*c*) If relevant, consider translating estimates of relative risk into absolute risk for a meaningful time period	N/A
Other analyses	17	Report other analyses done—eg analyses of subgroups and interactions, and sensitivity analyses	N/A
**Discussion**			
Key results	18	Summarise key results with reference to study objectives	8
Limitations	19	Discuss limitations of the study, taking into account sources of potential bias or imprecision. Discuss both direction and magnitude of any potential bias	10
Interpretation	20	Give a cautious overall interpretation of results considering objectives, limitations, multiplicity of analyses, results from similar studies, and other relevant evidence	10
Generalisability	21	Discuss the generalisability (external validity) of the study results	10
**Other information**			
Funding	22	Give the source of funding and the role of the funders for the present study and, if applicable, for the original study on which the present article is based	11

[a]Page numbers refer to the submitted manuscript document

STROBE, Strengthening the Reporting of Observational Studies in Epidemiology.

Table S2. Age adjusted incidence and mortality rates of esophageal cancer patients between 1975 and 2016

Time Period	Overall			
	Incidence Rate[1]		Mortality Rate[1]	
	Rural	Urban	Rural	Urban
1975 - 1989	4.66	2.39	3.89	1.86
1990 - 2000	5.23	2.54	4.39	1.97
2001 - 2010	5.88	2.97	4.61	2.19
2011 - 2016	6.40	3.07	4.99	2.24

[1] Age adjusted incidence and mortality rates are per 100,000 people

Age adjusted overall incidence and mortality rates per 100,000 people in urban and rural areas are shown.

Table S3. Attributable risk percentage and population attributable risk percent between 1975 and 2016 for esophageal cancer incidence.

YEAR	Population attributable risk percent	Attributable risk percentage (95% CI)
1975	3.98942	54.52 (28.10, 80.94)
1976	4.77311	54.19 (30.36, 78.01)
1977	2.32555	41.28 (6.71, 75.85)
1978	2.8153	40.04 (9.75, 70.33)
1979	3.04663	46.15 (17.51, 74.79)
1980	3.11128	50.53 (22.07, 78.99)
1981	1.39428	30.02 (-10.71, 70.74)
1982	2.98689	57.05 (27.54, 86.55)
1983	2.58241	44.13 (12.64, 75.61)
1984	3.82566	48.57 (23.08, 74.07)
1985	2.81821	44.02 (15.88, 72.17)
1986	3.75946	50.23 (26.27, 74.19)
1987	3.17514	42.02 (15.40, 68.64)
1988	6.57157	60.72 (42.33, 79.12)
1989	3.63561	50.19 (24.40, 75.99)
1991	5.87596	60.10 (40.44, 79.75)
1992	6.51571	61.90 (44.16, 79.64)
1993	3.97054	50.16 (27.65, 72.68)
1994	2.73489	37.50 (11.39, 63.60)
1995	5.74299	54.89 (35.60, 74.18)
1996	4.42236	47.63 (26.97, 68.28)
1997	4.90159	49.65 (29.72, 69.59)
1998	3.91093	46.05 (24.55, 67.55)
1999	3.37158	45.41 (23.45, 67.36)
2000	6.2944	54.74 (38.67, 70.82)
2001	4.95406	50.14 (32.20, 68.09)
2002	4.39056	47.61 (29.00, 66.22)
2003	5.22068	48.55 (31.59, 65.51)
2004	4.6825	47.89 (31.13, 64.65)
2005	5.21185	52.27 (35.21, 69.32)
2006	4.26464	46.91 (28.75, 65.07)

2007	3.56445	39.41 (20.29, 58.53)
2008	5.81229	49.49 (34.44, 64.55)
2009	6.41988	55.43 (41.00, 69.86)
2010	6.06377	53.24 (38.32, 68.17)
2011	5.97584	54.31 (39.59, 69.03)
2012	6.9834	54.92 (41.04, 68.80)
2013	6.24818	54.75 (40.81, 68.69)
2014	6.1338	51.16 (36.33, 65.98)
2015	3.96502	44.20 (27.15, 61.25)

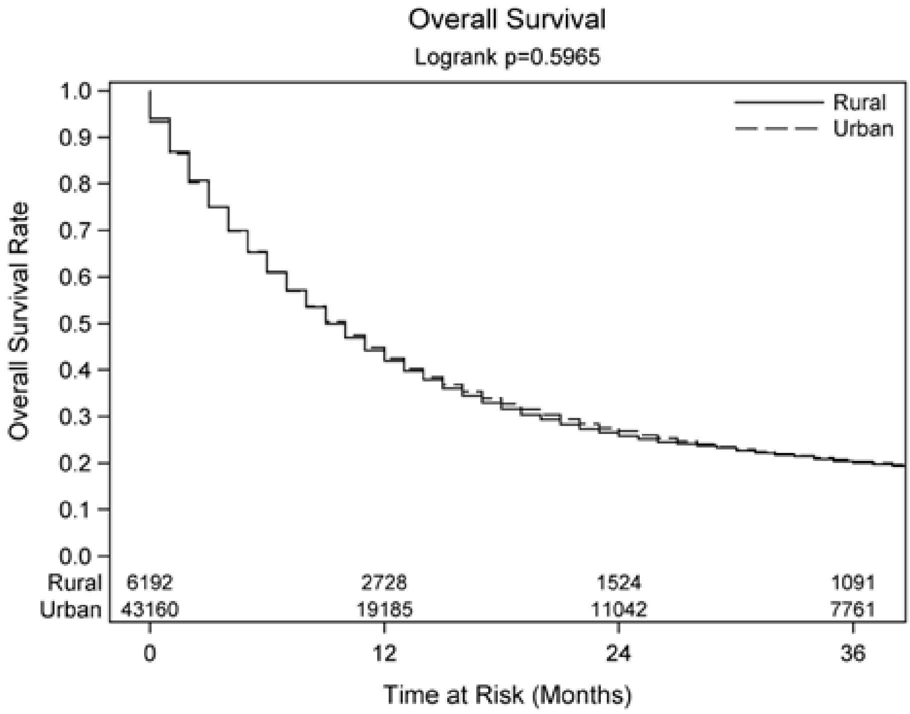

Figure S1. Kaplan Meier Survival Plot for Overall Survival between urban and rural oesophageal cancer patients.

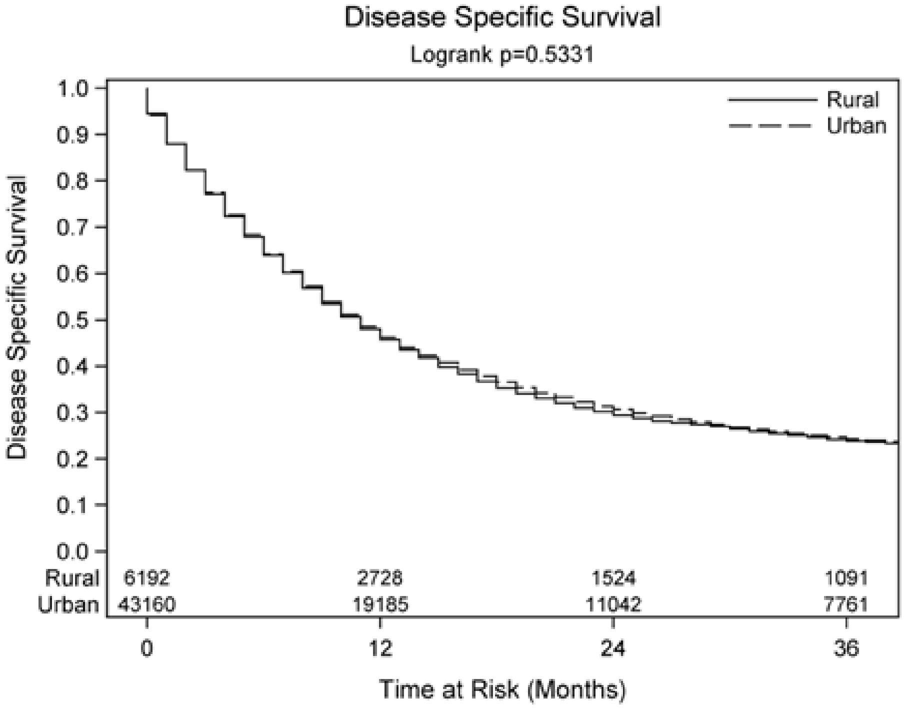

Figure S2. Kaplan Meier Survival Plot for Disease Specific Survival between urban and rural oesophageal cancer patients
